# Electrochemical Corrosion Behaviour of X70 Steel under the Action of Capillary Water in Saline Soils

**DOI:** 10.3390/ma15103426

**Published:** 2022-05-10

**Authors:** Jianjian Wei, Bin He, Yongxiang Feng, Lifeng Hou, Pengju Han, Xiaohong Bai

**Affiliations:** 1 College of Civil Engineering, Taiyuan University of Technology, Taiyuan 030024, China; weijianjian0687@link.tyut.edu.cn (J.W.); fengyongxiang0702@link.tyut.edu.cn (Y.F.); hanpengju@tyut.edu.cn (P.H.); bxhong@tyut.edu.cn (X.B.); 2 College of Materials Science and Engineering, Taiyuan University of Technology, Taiyuan 030024, China; houlifeng@tyut.edu.cn

**Keywords:** capillary effect, water-salt migration, electrochemical impedance, polarization curve

## Abstract

In this paper, the electrochemical corrosion behavior of X70 steel in saline soil under capillary water was simulated by a Geo-experts one-dimensional soil column instrument. A volumetric water content sensor and conductivity test were used to study the migration mechanism of water and salt (sodium chloride) under the capillary water. The electrochemical corrosion behavior of the X70 steel in the corrosion system was analyzed by electrochemical testing as well as the macroscopic and microscopic corrosion morphology of the steel. The test results showed that the corrosion behavior of X70 steel was significantly influenced by the rise of capillary water. In particular, the wetting front during the capillary water rise meant that the X70 steel was located at the three-phase solid/liquid/gas interface at a certain location, which worsened its corrosion behavior. In addition, after the capillary water was stabilized, the salts were transported with the capillary water to the top of the soil column. This resulted in the highest salt content in the soil environment and the most severe corrosion of the X70 steel at this location.

## 1. Introduction

In May 2017, China’s National Development and Reform Commission and the National Energy Agency jointly issued a “medium- and long-term oil and gas pipeline network plan”, which clearly stated that China’s oil and gas pipeline network would reach 240,000 km by 2025. However, the corrosion of buried metals is a major problem around the world. Metal pipelines in soil environments can form corrosive primary batteries, resulting in the electrochemical dissolution of the metal [1,2,3,4,5]. This problem is particularly prominent in saline soil [6,7,8,9].

Many domestic and international scholars have carried out a great deal of research into the corrosion of metals. Muhammad et al. performed a preliminary experimental study on the factors influencing the corrosion of buried pipes in corrosive soils [10]. Moreover, several studies have demonstrated that many factors significantly affect steel corrosion in saline soils. These factors include soil moisture content [11], pH [12,13,14], ion concentration [15,16,17], and the presence of microorganisms [18,19,20,21,22,23]. Han et al. found that corrosion rates increased as the pH of contaminated silty soil decreased [24]. Wang et al. showed that the corrosion rate of pipeline steel steadily increased with increasing AC density [25]. Zhang et al. reported that temperature, dissolved oxygen concentration, and pH had a significant effect on the corrosion current density I_corr_ of X70 steel [26]. Further, Khalaj and Pouraliakbar et al. investigated the correlation of passivation current density and potential using chemical composition and corrosion cell characteristics in HSLA steels by means of an artificial neural network approach, and the influence of physical and chemical parameters on toughness of pipeline steel is simulated by high-precision model. This provides a basis for the application and corrosion monitoring of pipeline steel [27,28]. A significant amount of research has also been reported by domestic and international scholars on the effects of capillary action [29,30,31,32,33,34,35,36,37,38,39,40]. Rad and Shokri studied the influence of evaporation from porous media of salt solutions by the interaction between the transport properties of the porous media, the thermodynamics of the evaporating solution, and the environmental conditions [41]. Moreira et al. explored the hydraulic and solute transport properties in unsaturated soils through small-scale indoor seepage tests [42]. Henry and Holtz showed that in areas of strong water evaporation, groundwater and water-soluble salts constantly migrated to the upper part of soil under capillary action [43].

Due to the continuous promotion of China’s West-East gas transmission project, X70 and X80 pipeline steel (which is used to transport oil and natural gas) has been produced in large quantities. Salts in saline soils (especially chlorine salts) and saline groundwater can chemically react with buried pipelines through capillary water action. This causes the corrosion of oil and gas pipelines. However, existing simulated corrosion environments rarely monitor changes in parameters such as soil moisture content during corrosion. Thus, the corrosion behavior and corrosion mechanisms of X70 steel in a dynamic environment with moisture and salt migration are not yet clear. The aim of this paper was to address this shortcoming. First, this paper investigated the effects of moisture and sodium chloride transport on the corrosion behavior of X70 steel in saline soils by volumetric water content sensors and conductivity tests. Second, the electrochemical corrosion behavior of X70 steel in saline soils under the action of capillary water was studied using electrochemical testing techniques as well as macro and micro corrosion morphology. Finally, the corrosion patterns of X70 steel under capillary action and its corrosion mechanism were revealed according to the water-salt migration law during capillary action.

## 2. Materials and Methods

### 2.1. Materials

#### 2.1.1. X70 Steel Specimens

X70 pipeline steel specimens with dimensions of 15 mm × 15 mm × 2 mm were designated as the working electrode. The X70 steel used in the test was supplied by Baoshan Iron & Steel Co in Shanghai, China. The main chemical composition of X70 steel is shown in Table 1.

**Figure 1:**
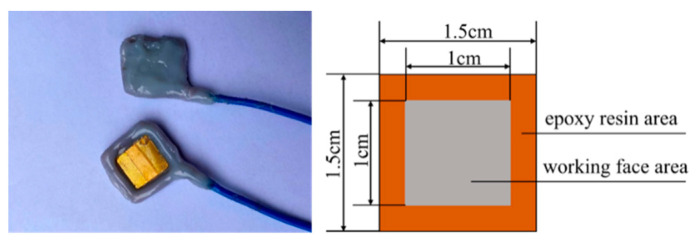
Schematic diagram of the physical dimensions and size of the X70 steel sample.

The X70 steel specimens were sanded step by step with 800–1500 grit sandpaper and then polished with 2000 grit sandpaper. These specimens were then placed in pure water for ultrasonic cleaning. The clean specimens were removed and air-dried for use. A working surface of 10 mm × 10 mm was left on the surface of the specimens, and their thickness position was wrapped with copper wire. The non-working surfaces were covered with epoxy resin, as shown in Figure 1.

#### 2.1.2. Soil Specimens

**Figure 2:**
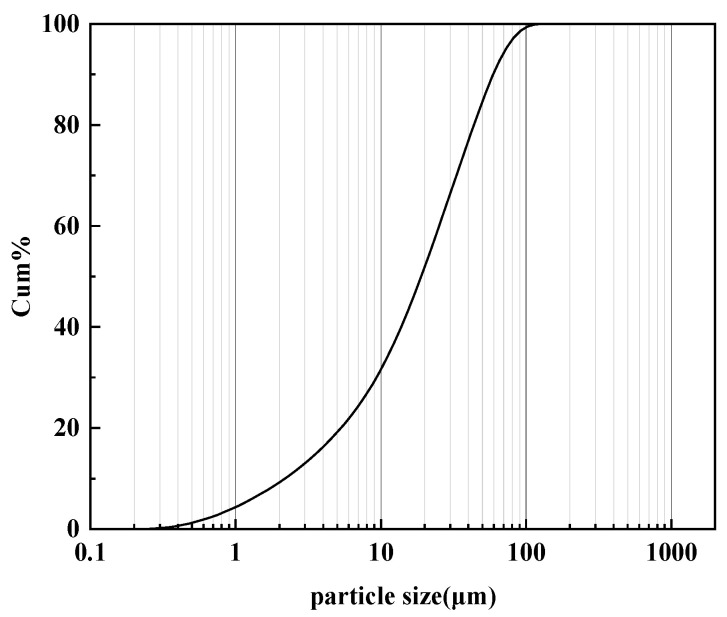
Particle gradation curve of the test soil.

**Figure 3:**
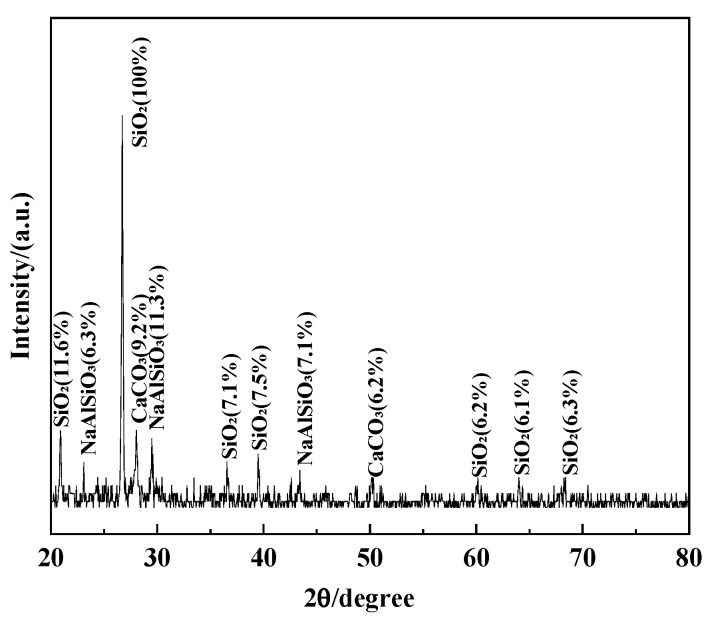
XRD spectrum of soil.

The test soil was taken from the foundation soil near a buried oil and gas pipeline in Dongshan, Taiyuan, Shanxi Province, China. The soil particle size distribution was analyzed using a BT-9300S laser particle size distribution meter, as shown in Figure 2. This analysis shows that the soil was classified as silty clay loam. BT-9300S is manufactured by Dandong Baxter Instruments Ltd. in Dandong, Liaoning Province, China. The basic physical parameters of the soil are listed in Table 2. The XRD pattern of this soil is shown in Figure 3. This diffraction pattern shows that the main component of the powdered soil was SiO_2_. The soil was naturally dried and sieved through a 2 mm sieve.

To simulate saline soil environments with different salt contents, NaCl was added to the soil samples, which were then well mixed by hand. The relevant ratios used for the tests are shown in Table 3. The mass moisture content of all of the saline soil specimens was controlled at 12.24% during testing.

### 2.2. Methods

**Figure 4:**
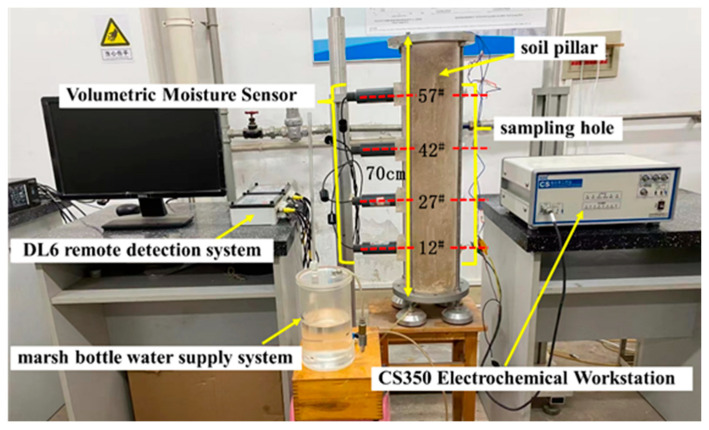
Geo-experts one-dimensional soil column instrument.

A Geo-experts one-dimensional soil column instrument was used for this test. This instrument consisted of a constant water supply system, a Plexiglass cylinder, and a volumetric water content sensor, as shown in Figure 4.

**Figure 5:**
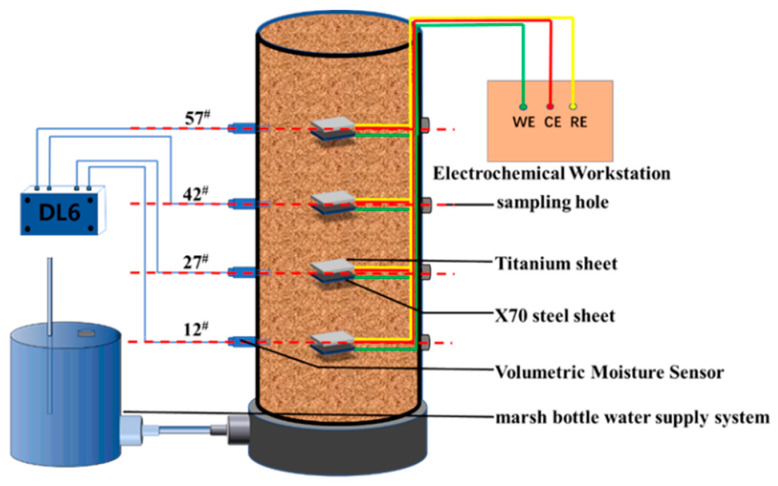
Experimental device.

To prepare each column, a uniform layer of petroleum jelly was first applied to the inside of the Plexiglass cylinder, which was fixed to the test base. Next, a layer of filter paper was laid on the bottom of the cartridge, and a 1 cm thick layer of standard sand was loaded on top of the filter paper to ensure an even rise in moisture. Finally, the configured soil sample was buried in layers by means of compaction. A fixed mass of soil sample was weighed and compacted to a thickness of 1 cm for each layer to ensure that the entire column had the same preset dry density. These steps were repeated to four different heights in the soil column: 12^#^, 27^#^, 42^#^, and 57^#^. Next, the X70 steel specimens and parallel titanium electrodes with the same dimensions were buried in the soil column. After the column was filled, a constant head water supply system was connected at the bottom of the column to simulate capillary water action. ML2x moisture sensors were placed at heights 12^#^, 27^#^, 42^#^, and 57^#^ on one side of the column to monitor the changes in the moisture content of the soil at those locations. As shown in Figure 5, the sampling holes were left on the other side of the column to facilitate the removal of soil samples for conductivity measurements. A certain amount of soil samples was taken out through the sampling hole at the specified time and then dried naturally. The natural air-dried soil samples were added with water at the ratio of 1:5 and centrifuged at 3000 r/min for 30 min under the condition of 20 °C ± 1 °C to obtain the extract. Subsequently, a DDSJ-307A conductivity meter was used to determine the conductivity of the extract at 25 °C ± 1 °C. DDSJ-307A is manufactured by Shanghai Yidian Scientific Instruments Co. in Shanghai, China. The principle of measuring conductivity is that when the two electrodes are inserted into the extraction solution, the resistance between the two electrodes can be measured. When the temperature is constant, the resistance value R is inversely proportional to the conductivity K, namely, R = Q/K. When the conductivity cell constant Q is known, the conductivity can be obtained by measuring the resistance of the extract. Three parallel samples were taken for each conductivity measurement, and the final average of three replicate conductivity measurements was taken.

In this experimental study, an electrochemical test method was used to investigate the electrochemical corrosion behavior of X70 steel during capillary water rise. Fourteen, 28, and 45 days were used as the ageing periods. A CS350 electrochemical workstation was used for the electrochemical tests. The X70 steel sheet specimens were used as the working electrode (WE), and the titanium electrode was used as the counter electrode (CE) and the reference electrode (RE). The relative hydrogen standard potential of the reference electrode at 25 °C is −1.75 V. The capillary water rise of the soil column was completed at 90 days. At this time, the steel sheet specimens and soil samples were removed. The soil samples were used to measure the soil matrix suction at different height positions using a dew point water potential meter. The microscopic morphology of the X70 steel specimens was observed by SEM and 3D contour scanning.

## 3. Results and Discussion

### 3.1. Water-Salt Migration Patterns under Capillary Water Action in Saline Soils

#### 3.1.1. Variation of Volumetric Water Content at Different Locations in the Soil Column

**Figure 6:**
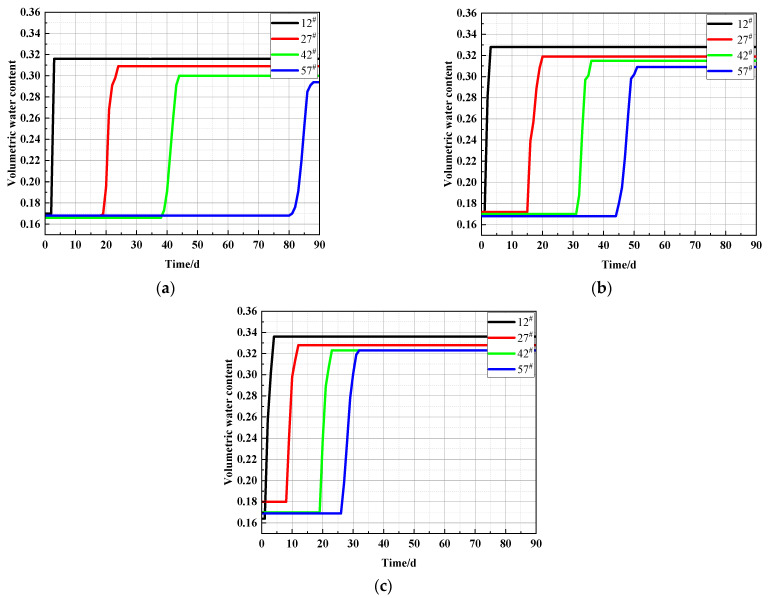
Variation of soil column volume water content with time for different NaCl contents: (**a**) 0%, (**b**) 0.3%, (**c**) 1.0% NaCl.

Figure 6a–c shows the variation of volumetric water content with time at different heights in soil columns with sodium chloride content of 0.0%, 0.3%, and 1.0%. The final stable water content of all three soil columns with different salt contents showed the same pattern, i.e., the final stable water content of the soil column gradually decreased from the bottom of the column to the top of the column. This was due to the soil water potential associated with the soil moisture. The water in soil is in static equilibrium when the potential energy of the water is equal at each point in the soil [44,45,46,47]. The soil water potential can be expressed as:

(1) $$\Psi =\Psi_{g}+\Psi_{p}+\Psi_{m}+\Psi_{s}+\Psi_{r}+\ldots$$

where:

Ψ—soil water potential, J/kg;

Ψ*_g_*—gravitational potential, J/kg;

Ψ*_p_*—pressure potential, J/kg;

Ψ*_m_*—matric potential, J/kg;

Ψ*_s_*—solute potential, J/kg;

Ψ*_r_*—temperature potential, J/kg.

In this test, the soil pores were connected to the atmosphere. Thus, no additional pressure was present. The pressure potential at each point in the soil column at this time was:

(2) $$\Psi_{p}=0$$

The experiment was carried out at room temperature. Thus, there was almost no temperature difference caused by a temperature field. The temperature potential at this time was:

(3) $$\Psi_{r}=0$$

The magnitude of the gravity potential depended on the height of the water position. After selecting the reference plane, the gravity potential of soil moisture per unit weight with height *h* was described as:

(4) $$\Psi_{g}=h$$

In summary, the soil water potential per unit mass of soil moisture was:

(5) $$\Psi =h+\Psi_{m}+\Psi_{s}$$

Because the total soil water potential at each point of the soil column was zero in the equilibrium state:

(6) $$\Psi_{m}=-\left(h+\Psi_{s}\right)$$

As the capillary water rose, the ions in the soil column gradually moved towards the top of the column along with the moisture in the soil. In this test, the ion concentration in the soil column at the point of final water stabilization showed a gradual increase from the bottom to the top of the column. This ion concentration gradient meant that the solute potential also increased with increasing column height.

According to Equation (6), with increasing soil column height, the sum of gravitational potential h and solute potential Ψs of the soil moisture per unit weight increased; the matric potential decreased; the matric suction increased; and the water content decreased. This explained why the final stable water content of the soil column gradually decreased from the bottom to the top of the soil column. As shown in Figure 6, the curve of water content shifted to the left of the axis as the NaCl content in the soil column increased, suggesting that the presence of NaCl had a beneficial effect on the early capillary water rise rate [48]. There were two reasons for this trend. On the one hand, the salt in saline soil dissolves in water and increases the surface tension of the water. On the other hand, NaCl is very soluble in water, and crystal precipitation is not sensitive to changes in temperature. Thus, NaCl is not a significant channel blocker for water migration.

#### 3.1.2. Variation of Conductivity at Different Locations in the Soil Column

**Figure 7:**
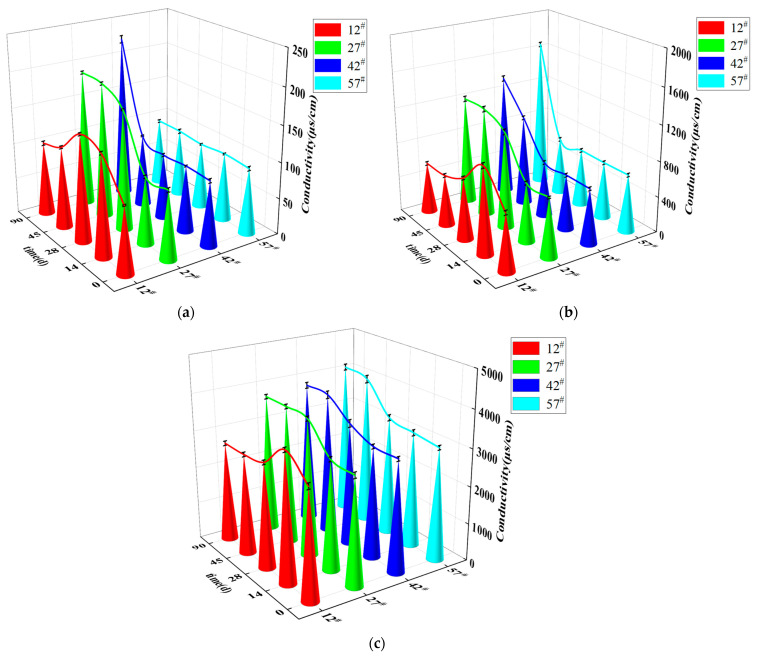
Variation of soil column conductivity with time for different NaCl contents: (**a**) 0%, (**b**) 0.3%, (**c**) 1.0% NaCl.

The pores between soil particles were interconnected, which led to the formation of capillary channels in the soil. These were the channels that allowed the capillary water to rise in unsaturated soil. The main driver of water and salt migration in the soil was the presence of free and weakly bound water in the soil. Free water continuously rose by capillary action, while weakly bound water was transferred from the thicker to the thinner part of the water film by external forces. This led to the migration of water, and, after NaCl dissolved in the water, the NaCl migrated with the water. Figure 7a–c shows the variation of soil conductivity with age at four different heights in the soil column under NaCl concentrations of 0%, 0.3%, and 1.0%, respectively.

After 90 days of ageing, the capillary water in the soil column with 0% NaCl content was stabilized. However, no significant increasing conductivity trend was visible at height position 57^#^, as shown in Figure 7a. This indicated that the transport of salts in the soil lagged relative to the transport of water. The rise of capillary water in the soil columns containing 0.3% and 1.0% NaCl also reached steady state after 90 days, as shown in Figure 6. As shown in Figure 7, the change in conductivity after 90 days of ageing in the 0.3% and 1.0% NaCl columns showed the same pattern: the conductivity gradually increased from the bottom to the top of the column as the capillary water in the column reached a stable state. In addition, the conductivity of the soil columns with 0.3% and 1.0% salinity showed a pattern of increasing conductivity with age at locations 27^#^, 42^#^, and 57^#^. These two phenomena occurred because sodium chloride in the soil column continuously accumulated at the higher part of the soil column with increasing capillary water.

As shown in Figure 7, the soil electrical conductivity at height 12^#^ in the three soil columns with different NaCl content first increased and then decreased with increasing age up to 90 days. The salt ions at the bottom of the soil column rose with the migration of water to height position 12^#^, which led to temporarily higher conductivity at this position. After a long period of moisture migration, some of the salt at position 12^#^ migrated with the moisture to a higher position in the column. This led to lower conductivity at this position.

### 3.2. Polarization Curves of X70 Steel in Saline Soils under Capillary Action

#### 3.2.1. Effect of Different Salt Content on the Polarization Curve of X70 Steel under Capillary Action

**Figure 8:**
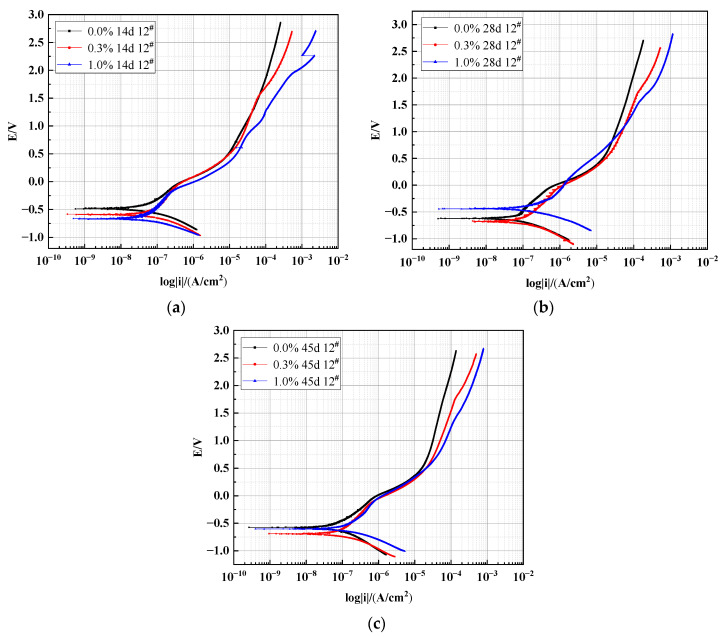
Polarization curve of X70 steel at 12^#^ height: (**a**) 14 days, (**b**) 28 days, and (**c**) 45 days.

The polarization curves of the X70 steel embedded in height 12^#^ of the 0%, 0.3%, and 1.0% NaCl soil columns after 14 days, 28 days, and 45 days are shown in Figure 8. The test results were processed using CView2 fitting software to obtain the electrochemical corrosion parameters of the X70 steel, and the results are listed in Table 4.

The top half of each polarization curve represented the anodic process of a corrosion cell, where an oxidation reaction took place. This oxidation reaction led to the metal dissolving, and the dissolved metal entered the corrosion system as ions. The lower half of the polarization curves represented the cathodic process of the corrosion cell, where the reduction reaction occurred. This reduction reaction was mainly controlled by the diffusion of the dissolved oxygen limit [49]. After 14 days, significant passivation was visible in the anodic branch of the X70 steel polarization curve for the contaminated soil containing 1.0% NaCl [50]. When this metal was passivated, the anode dissolution process no longer obeyed Fertal’s law, and the anode electrode dissolution rate decreased. This was probably due to the formation of an inner corrosion product film layer that prevented the diffusion of ferrous ions. By comparing the corrosion rate of X70 steel in the soil corrosion environment of 1.0% sodium chloride content in Table 4 at 14 days, 28 days, and 45 days, it can be seen that the corrosion rate of 14 days is 0.9191 × 10^−3^ mm/a, which is significantly less than 1.3049 × 10^−3^ mm/a of 28 days and 2.1896 × 10^−3^ mm/a of 45 days. Combined with the passivation of the polarization curve of X70 steel at 14 days mentioned above, I speculate that the fact that the corrosion rate of X70 steel at 14 days is less than at 28 days and 45 days is related to the passivation of X70 steel at 14 days. The presence of this metal passivation film resulted in a significantly lower corrosion rate at 14 days compared with 28 days and 45 days in the contaminated soil with 1.0% NaCl [51].

As shown in Figure 8, the X70 steel polarization curves shifted to the right to varying degrees as the concentration of NaCl in the contaminated system increased. This indicated that the presence of salt in the contaminated system played a role in accelerating the electrochemical process of X70 steel corrosion. The salt in the pore solution promoted the ion exchange capacity of the electrode interface, which accelerated the corrosion rate. Due to the presence of chlorine salts, the self-corrosion potentials of the X70 steel specimens all shifted to varying degrees in the negative direction. A more negative self-corrosion potential indicated a greater tendency to corrode. As shown in Figure 8a, the self-corrosion potential Ecorr of the X70 steel sheet in the contaminated soil with 1.0% NaCl content at 14 days exhibited the most negative value. Thus, the corrosion thermodynamic trend of this specimen was high, and it was prone to corrosion.

It can be seen from Table 4 that the polarization resistance Rp of position 12^#^ at three corrosion ages in the corrosion environment decreases with the increase of NaCl content. This is because the dielectric constant of the soil increases as the sodium chloride content increases [52]. In addition, the results of the study show that the polarization resistance is inversely proportional to the corrosion rate of the metal [53]. This means that the corrosion rate of X70 steel increases with the increase of sodium chloride content in soil corrosion environment. In addition, this conclusion can be verified by the corrosion current density and corrosion rate in Table 4. The results reported in Table 4 show that the corrosion current density Icorr and corrosion rate of the X70 steel were enhanced by higher NaCl content in the soil after 14, 28, and 45 days. This further indicated that the presence of Cl^−^ had increased the corrosion rate of X70 steel. At 14 days, the corrosion current density of the X70 steel in the three different salt-contaminated soils showed an accelerating trend with increasing NaCl content. However, the corresponding corrosion current density was still in the same order of magnitude at this time. As the corrosion process continued, the corrosion current density of the X70 steel initially increased by one order of magnitude in the soil column containing 1.0% NaCl at 28 days and by one order of magnitude in the soil column containing 0.3% NaCl at 45 days. This demonstrated that, within a certain chloride ion concentration range, higher chloride ion concentrations led to a more rapid increase in the X70 steel corrosion rate. This was because higher Cl^−^ concentrations in these tests led to a more destructive corrosion product film on the surface of the steel specimens. Cl^−^ ions were able to penetrate the corrosion product film produced by the pit corrosion of the metal in contact with the corrosive environment. This increased the surface area and the current concentration. Eventually, this corrosion product film was unable to continue protecting the X70 steel sample, leading to a sharp increase in corrosion rate.

As shown in Table 4, the corrosion rate of the X70 steel in a soil column with 0.3% NaCl showed a decreasing and then increasing trend with age. The surface of this steel tended to form a denser oxide film that prevented corrosive ions in the soil from coming into contact with the steel surface. This led to a reduction in corrosion current density and corrosion rate [54]. As the corrosion process continued, this corrosion product layer on the steel specimen surface gradually thickened, but the outermost layer of corrosion products was loose and porous. The presence of Cl^−^ eroded this corrosion product film, allowing corrosion to continue.

#### 3.2.2. Effect of Different Heights on the Polarization Curve of X70 Steel under Capillary Action

**Figure 9:**
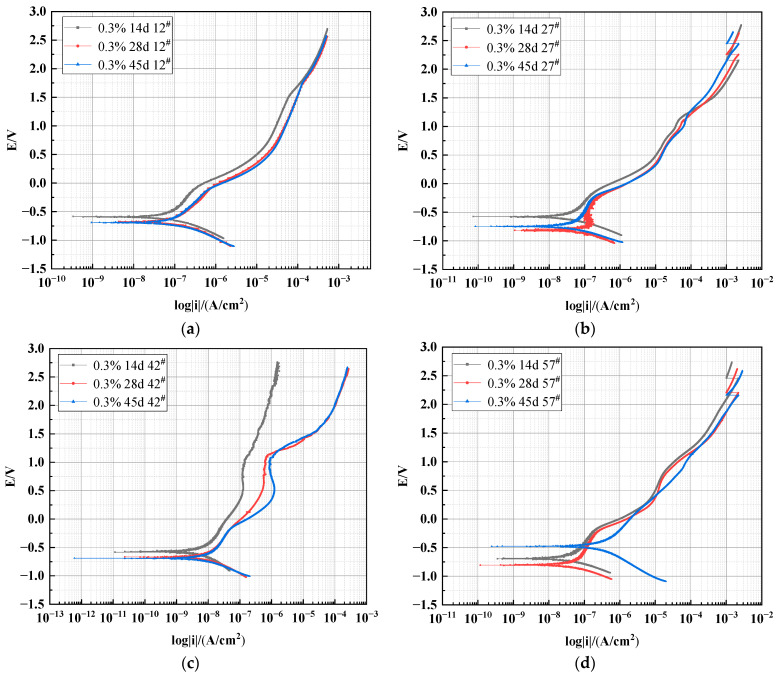
Polarization curves of X70 steel buried in 0.3% NaCl soil column: (**a**) 12^#^, (**b**) 27^#^, (**c**) 42^#^, and (**d**) 57^#^.

The polarization curves of X70 steel specimens buried at heights of 12^#^, 27^#^, 42^#^, and 57^#^ in 0.3% NaCl soil columns for 14, 28, and 45 days are shown in Figure 9. The test results were processed using CView2 fitting software to obtain the electrochemical corrosion parameters of X70 steel, and the results are listed in Table 5. As shown in Figure 9a, the corrosion rate of X70 steel at location 12^#^ in the soil column was greater at 14 days. This was due to the accumulation of sodium chloride at the bottom of the soil column as the capillary water rose to location 12^#^. This higher sodium chloride concentration contributed to the corrosion of the X70 steel. Table 5 shows that the corrosion potential of the X70 steel buried at position 12^#^ in the soil column decreased with time. This was because, in the early stage of corrosion, corrosion preferentially occurred on areas of the steel sheet with low surface potential. Because the yield of these corrosion products was low, no protective film was formed on the surface of the steel sheet. Therefore, the X70 steel was further corroded, and the corrosion potential decreased with time.

Due to the rise of the capillary water at position 27^#^ in the soil column at 14 days, the X70 steel specimen in this location was in a corrosive environment in which the water content was high and the contact area between the corrosive medium and X70 steel was large. This resulted in the homogeneous formation of double-layer capacitance on the metal surface and, therefore, a more homogeneous and dense oxide protection layer. Consequently, a lower corrosion rate was achieved. As shown in Table 5, the corrosion current density of the X70 steel at height 27^#^ in the soil column at 28 days was 1.0495 × 10^−7^ A/cm^2^. This was an order of magnitude higher than the corrosion current density of the height 27^#^ X70 steel at 14 or 45 days. This trend was attributed to the migration of chloride salts in the contaminated soil with the rise of capillary water and the accumulation of salts at this location. The above conclusions can also be easily obtained by comparing the changes in polarization resistance. At 28 days, the polarization resistance at position 27^#^ in the soil column is significantly lower than at the other two ages. This is attributed to the accumulation of salts at 27^#^, which increases the dielectric constant at this location, making ion exchange in the corrosive environment more likely to occur and ultimately accelerating the corrosion rate.

Figure 9b shows that the polarization curve of X70 steel at height position 27^#^ of the soil column at 28 days was more negative than the other two polarization curves. This demonstrated that the self-corrosion potential of this specimen at 28 days was more negative, which was an indication that the thermodynamic corrosion trend of this X70 steel specimen was greater and that this specimen was more prone to corrosion. At 45 days, NaCl still accumulated at this location, but the transport path for oxygen was blocked, because this location was below the wetting front. This resulted in a reduced corrosion rate [55]. The X70 steel specimens at height positions 42^#^ and 27^#^ showed the same corrosion rate variation trend.

At 14 and 28 days, the capillary water had not yet reached position 57^#^, although there was sufficient oxygen for the corrosive reaction at this location due to the low water content. This was not conducive to the formation of microscopic corrosion cells. Therefore, the corrosion rate of X70 steel in the soil at this position was lower at 14 or 28 days than at 45 days. At 45 days, the rising capillary water wetting front was located at position 57^#^. Thus, the X70 steel specimen at this location was at the junction of the solid-liquid-gas phase. At this time, the soil column 57^#^ position exhibited both high water content and sufficient oxygen content. This was extremely conducive to the electrochemical corrosion reaction, and so the corrosion rate reached its maximum value at this time.

### 3.3. Electrochemical Impedance Characteristics of X70 Steel in Saline Soils under Capillary Action

#### 3.3.1. Effect of Varying Salt Content on the Impedance Characteristics of X70 Steel under Capillary Action

**Figure 10:**
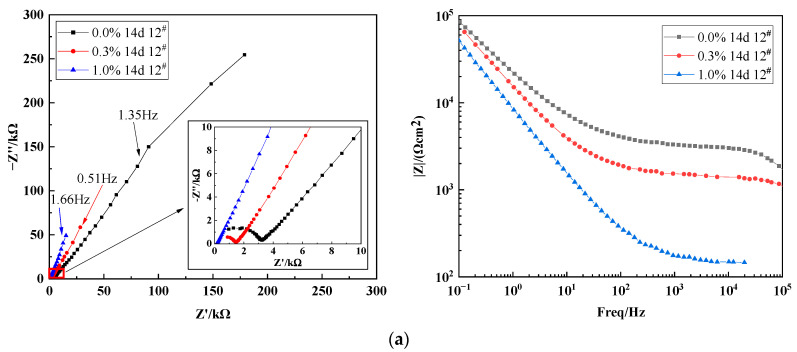

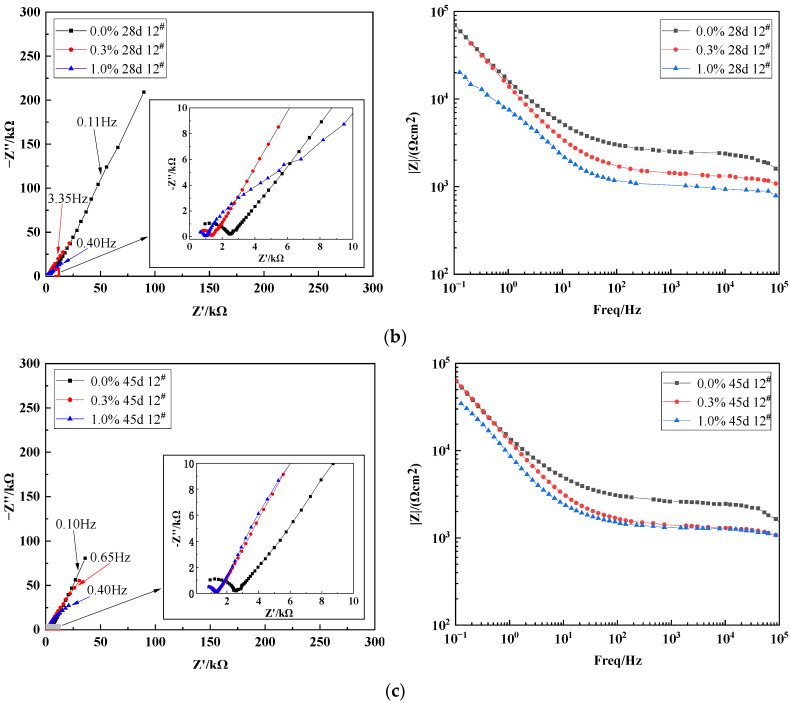
Nyquist and Bode plots of X70 steel at 12^#^ height of soil column: (**a**) 14 days, (**b**) 28 days, and (**c**) 45 days.

Figure 10 shows Nyquist and Bode plots for the X70 steel buried in 0.0%, 0.3%, and 1.0% NaCl soil columns at height 12^#^ for 14 days, 28 days, and 45 days. These electrochemical impedance profiles reflected the capacitive process in the high-frequency region, the corrosion-dissolution process in the medium-frequency region, and the diffusion process in the low-frequency region. The Nyquist diagrams showed that the impedance spectrum of X70 steel was a double arc with two time constants and a straight line superimposed in the low-frequency region. The diffusion tail arc in the low-frequency region at an angle of 45° to the real axis indicated that the corrosion process at the soil–steel interface was dominated by the mass transfer diffusion process. This was due to the large number of fine sticky particles adsorbed at the soil–electrode interface in this test soil corrosion system. The presence of these clay particles affected the soil–electrode interface and the pore channel structure between the soil particles. This affected the flow path of the pore electrolytes through the contaminated soil system. Ultimately, the charge transfer process during electrochemical corrosion was not directly achieved through the channels of the aqueous solution in the pores. This led to diffuse impedance, which was visible in the EIS results. In addition, the mass transfer diffusion during corrosion was affected by the different geometric properties of the soil–electrode contact surface, especially the roughness of this interface. This resulted in a possible deviation of the diffusion line in the low-frequency region of the Nyquist plots from the direction of the real axis at an angle of 45° [56]. The radius of the high-frequency capacitive arc in the Nyquist plots of the X70 steel at 14, 28, and 45 days for different NaCl concentrations decreased with increasing Cl^−^ concentration. That is, the resistance value decreased, and the corrosion rate increased, with increasing Cl^−^ concentration. The impedance modulus in the Bode plots decreased with increasing Cl^−^ concentration, and the corrosion rate also tended to increase with increasing Cl^−^ concentration [57]. This was consistent with the polarization curve analysis.

**Figure 11:**
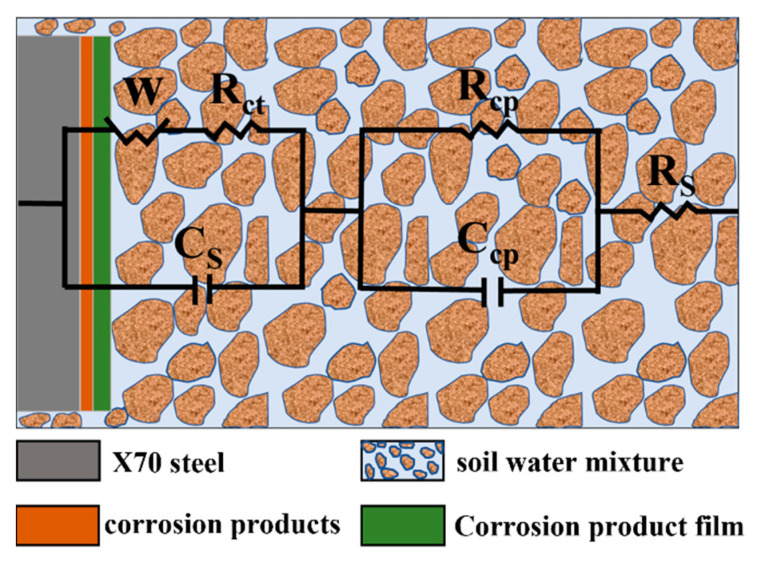
Equivalent circuit diagram of X70 steel in soil.

The equivalent circuit models for the X70 steel specimens in the soil columns with 0.0%, 0.3%, and 1.0% chloride content at height 12^#^ for 14 days, 28 days, and 45 days were fitted by ZView2 software, as shown in Figure 11. The electrochemical parameters in the equivalent circuit models above are shown in Table 6. The parameter R_s_ represents the soil resistance. The parameters obtained by fitting showed that the soil resistance R_s_ decreased with increasing NaCl content in the soil. This was because a higher NaCl concentration lowered the dielectric constant of the soil and enhanced the soil conductivity, which enhanced the corrosion process. R_ct_ is the charge transfer resistance, which characterizes the resistance to charge migration. The fitted parameters show that the R_ct_ value decreased with increasing NaCl content at 14, 28, and 45 days. This was because higher NaCl content in the soil column led to a higher concentration of conductive ions in the aqueous solution of the soil pores and, therefore, more corrosive soil. Charge transfer resistance was negatively correlated with the corrosion rate. Thus, the corrosion rate of the X70 steel gradually increased as the sodium chloride content increased, which was consistent with the polarization curve analysis. The fitted result parameters showed that the charge transfer resistance R_ct_ was significantly lower at 45 days in the soils with 0.3% and 1.0% NaCl compared with 14 or 27 days in the same soils. This indicated that the corrosion rate of X70 steel significantly increased at 45 days, which was also consistent with the polarization curve analysis. R_cp_ is the corrosion product resistance, which indicates the hindering effect of corrosion products on the ion transport migration process. With increasing corrosion time, R_cp_ showed a trend of first decreasing and then increasing. This meant that as the corrosion time increased, the corrosion product film formed during the corrosion process was destroyed by the strong penetration of Cl^−^, which reduced resistance to ion migration. Subsequently, as the corrosion process continued, corrosion products were continually generated, which impeded the reaction and led to a higher R_cp_ value. C_cp_ represents the corrosion product capacitance. The C_cp_ values of the X70 steel in the 1.0% NaCl soil column at 14 days were significantly higher by several orders of magnitude compared with other ages and other NaCl contents. This indicated that the corrosion product film was a greater barrier to ion transport migration at this time. After 14 days of ageing, the surface of the X70 steel produced a passivated corrosion product film that significantly hindered the ion transport migration process. C_s_ represents the characteristic bilayer capacitance of the clay particles at the soil–steel interface. As shown in Table 6, the value of C_s_ increased with increasing NaCl content in the corrosion system. This was because the ion concentration in the corrosion system affected the distribution of particles at the soil–steel interface. A higher concentration of electrolyte in the droplets on the surfaces of the clay particles led to a tighter double electric layer and smaller thickness. In turn, this led to a greater C_s_ value. W stands for Weber impedance Warburg.

The total impedance of the equivalent circuit:

(7) $$Z=R_{s}+\frac{1}{j\omega C_{cp}+\frac{1}{R_{cp}}}+\frac{1}{j\omega C_{s}+\frac{1}{R_{ct}+\left(1-j\right)\frac{\sigma }{\sqrt{\omega }}}}$$

where:

*Z*—total impedance, Ω·cm^2^;

*R_S_*—soil resistance, Ω·cm^2^;

*R_cp_*—corrosion product resistance, Ω·cm^2^;

*C_cp_*—corrosion product capacitance, F·cm^−2^;

*C_s_*—characteristic bilayer capacitance, F·cm^−2^;

*R_ct_*—charge transfer resistance, Ω·cm^2^;

*j*—imaginary number, $j=\sqrt{-1}$;

*ω*—angular frequency, rad/s;

*σ*—Corrosion product film stress, Pa.

#### 3.3.2. Effect of Height on the Impedance Characteristics of X70 Steel under Capillary Action

**Figure 12:**
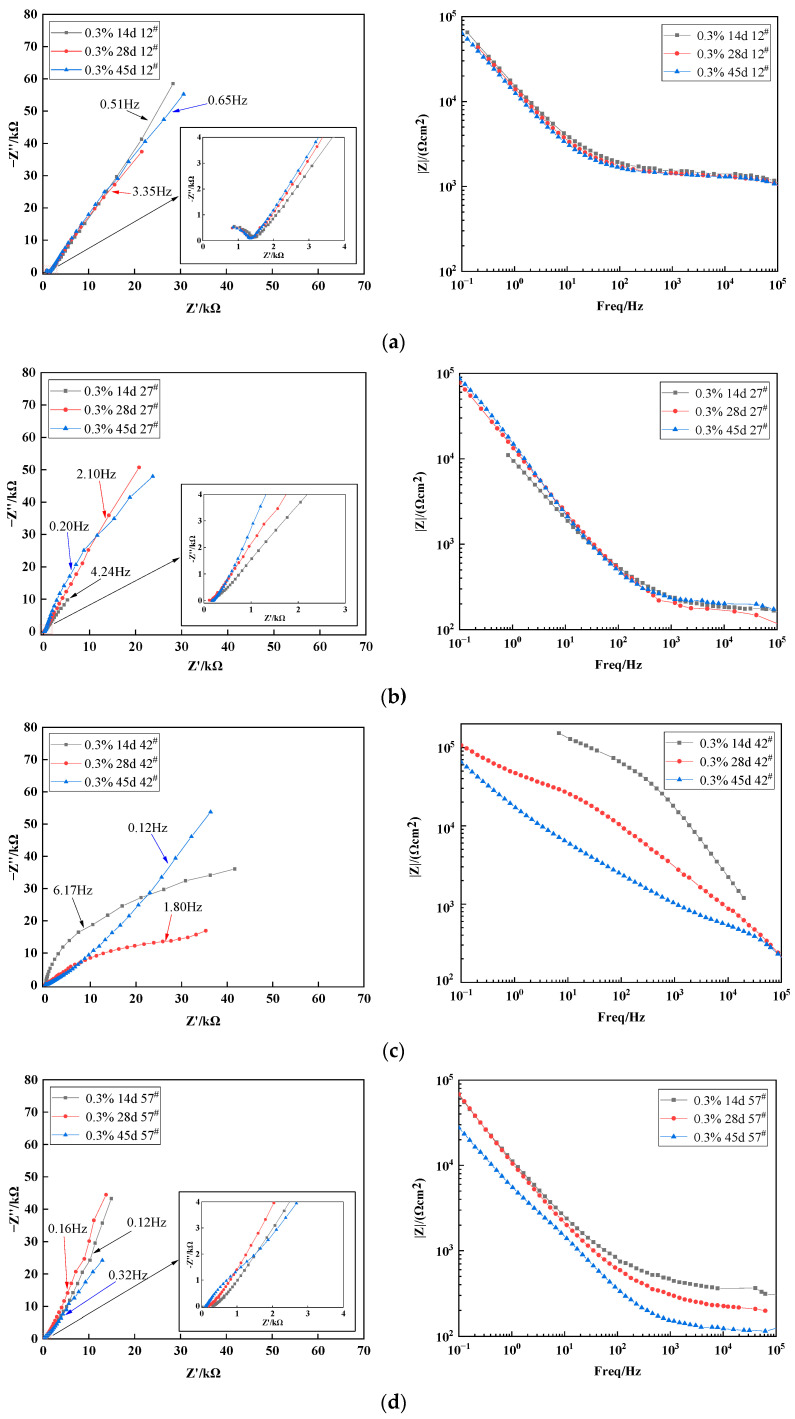
Nyquist and Bode plots of X70 steel in soil column with 0.3% sodium chloride: (**a**) 12^#^, (**b**) 27^#^, (**c**) 42^#^, and (**d**) 57^#^.

Figure 12 shows Nyquist and Bode plots of the X70 steel specimens buried in the 0.3% NaCl soil column at positions 12^#^, 27^#^, 42^#^, and 57^#^ for 14 days, 28 days, and 45 days. The radius of the capacitive arc in the 45-day high-frequency region of the Nyquist plots at position 12^#^ became significantly smaller, which was reflected in a corresponding reduction in impedance value [54]. The same pattern was observed in the Bode plots. The reduction in impedance enhanced the corrosion rate of the X70 steel specimens. One reason for this phenomenon was because the region with rising capillary water content became gradually more favorable for the formation of corrosion cells. The increase of solution led to good corrosion conditions for the transfer of the medium. The second reason for this phenomenon was that the outermost layer of the X70 steel corrosion products was not very dense. Thus, the strongly penetrating Cl^−^ ions could break through the corrosion product film and allow the corrosion reaction to continue. This led to a higher corrosion rate. The lower impedance modulus of the X70 steel at 45 days was clearly observed in the Bode plot for the corrosion of X70 steel at height 57^#^ in the soil column. The lower impedance modulus led to more aggressive corrosion. This was caused by the rise of the capillary water at 45 days. When the wetting front reached position 57^#^, the soil moisture content somewhat increased at this height. However, at this point, the solution had not yet fully occupied the capillary channels that transport oxygen between the soil particles. The higher water content and sufficient oxygen created favorable conditions for the corrosion reaction to occur. Therefore, the corrosion rate of X70 steel increased, which was also in line with the polarization curve analysis.

The corrosion reaction under the test conditions in this work was a highly complex reaction system. Thus, to further investigate the electrochemical corrosion characteristics of X70 steel, the equivalent circuit parameters of the X70 steel specimens buried at the height positions of 12^#^, 27^#^, 42^#^, and 57^#^ in the 0.3% NaCl content soil column for 14 days, 28 days, and 45 days were obtained by fitting with ZView2 software, as shown in Table 7. The equivalent circuit was the same as that shown in Figure 11. R_s_ is the soil resistance, and R_ct_ is the charge transfer resistance. Soil resistance R_s_ and charge transfer resistance R_ct_ were lower at 14 days at position 12^#^ of the soil column. This is because the salt collects at position 12^#^ as the capillary water rises. The accumulation of salts causes the dielectric constant of the soil to increase at this time, making it more conductive and reducing resistance. In addition, the 27^#^ and 42^#^ soil resistances R_s_ and charge transfer resistances R_ct_ were lower at 45 days for the same reason. R_ct_ is the charge transfer resistance, which characterizes the hindering effect on charge migration [58]. In the corrosive environment of the soil column at height positions 27^#^ and 42^#^, the charge transfer resistance R_ct_ showed a pattern of initially decreasing and then increasing. In the early stages of the corrosion reaction, the water content of the soil at these two locations gradually increased as the capillary water rose. This caused the dielectric constant of the corrosive soil system to decrease. Correspondingly, R_ct_ also decreased. The subsequent increase in R_ct_ was caused by the fine sticky particles in the corrosion system adsorbing at the steel–soil reaction interface, which prevents ion transport migration in the corrosion system. R_cp_ is the corrosion product resistance, and this term indicates the hindering effect of corrosion products on the ion transport migration process. C_cp_ is the corrosion product capacitance. Larger C_cp_ values were achieved at height positions 27^#^ and 57^#^. The C_cp_ values at these positions were between one and four orders of magnitude higher than the other two positions. This trend was an indication that the corrosion product film had a greater barrier effect on ion transport migration. The corrosion product film was associated with a passivation zone in the X70 steel polarization curve at both height positions. C_s_ is representative of the characteristic bilayer capacitance of clay particles at the soil–steel interface. Numerous fine clay particles were present in the corrosive environment of the X70 steel specimens in this test. The moisture in the soil penetrated the soil–steel contact interface when the X70 steel surface was in contact with the wet clay [59]. Electric double-layer capacitance was then formed at the solid–liquid two-phase interface between the X70 steel specimen and the moisture in the clay particles. A potential difference at this interface led to a potential difference on the surface of the X70 steel, which formed a primary cell that caused localized corrosion [60]. As shown in Table 7, as corrosion proceeded, the overall C_s_ value gradually increased. This was because water was continually replenished as the capillary water rose in the column, which led to its adsorption by the sticky particles. In this process, an increasing quantity of ions was adsorbed, and the double-layer capacitance stored an increasing amount of energy.

### 3.4. Macroscopic Corrosion Morphology of X70 Steel under Capillary Action in Saline Soils and the Corrosion Mechanism

#### 3.4.1. Macro and Micro Corrosion Profiles of X70 Steel

**Figure 13:**
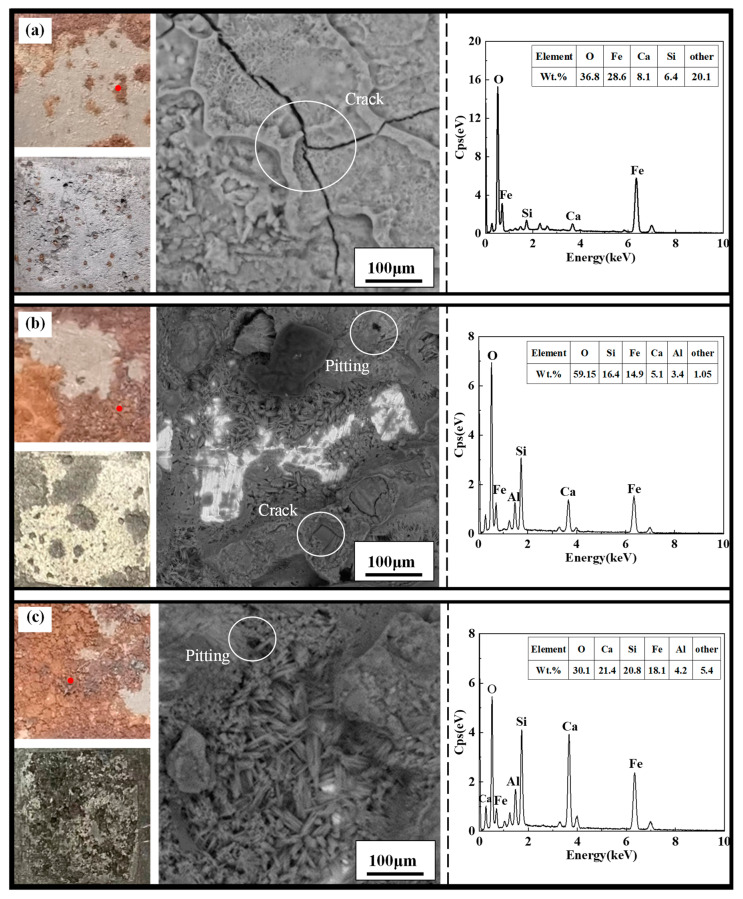
Macroscopic and microscopic corrosion morphology and elemental analysis results of X70 steel under capillary action in soil column at the height of 12^#^: (**a**) 0%, (**b**) 0.3%, (**c**) 1.0% NaCl.

The macroscopic and microscopic corrosion morphology and elemental analysis of the X70 steel samples located at height position 12^#^ in the soil columns with varying sodium chloride content at 90 days are shown in Figure 13. In Figure 13 the macroscopic corrosion pattern of X70 steel before and after descaling respectively and the microscopic corrosion pattern at the red dot in the macroscopic corrosion pattern are shown. Also, the graph on the right in Figure 13 shows the results of the elemental analysis obtained from the area of the microscopic corrosion pattern. In this paper, similar images are represented in the same way.

**Figure 14:**
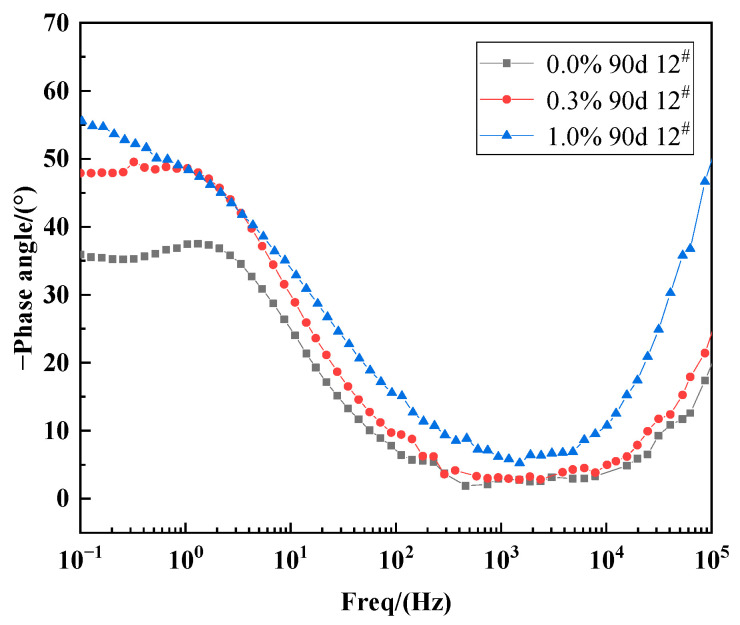
Curves of phase angle versus frequency for X70 steel at position 12^#^ in a corrosive soil environment with different sodium chloride content at 90 days.

**Figure 15:**
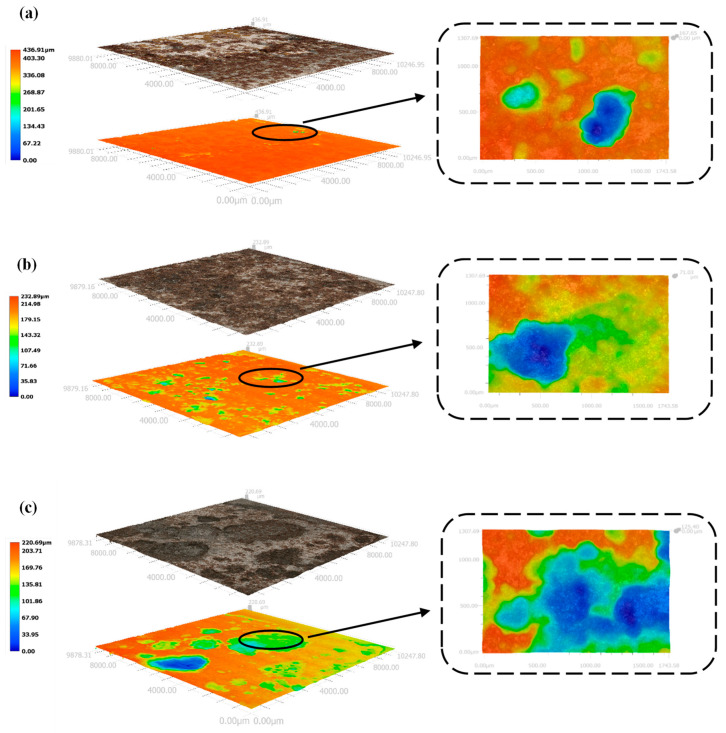
Three-dimensional morphological images of corrosion pits on the surface of X70 steel at the height of 12^#^ of soil column: (**a**) 0%, (**b**) 0.3%, (**c**) 1.0% NaCl.

As shown in Figure 13, the number of corrosion pits on the surface of the X70 steel gradually increased with increasing NaCl content for specimens located at the same height position in the soil column. Moreover, the area covered by corrosion products also increased in size with increasing NaCl content. The black-brown corrosion products (Fe_3_O_4_) and red-brown corrosion products (Fe_2_O_3_) were more tightly packed with increasing NaCl content, indicating deeper corrosion and a more severe reaction. At 90 days, the phase angle of X70 steel at 12^#^ position in soil corrosion environment with different sodium chloride content changes with frequency curve as shown in Figure 14. Since, in capacitive systems, the phase angle between current and tension is 90°, samples with phase angles close to this value are expected to better prevent the flow of electrolyte species [61,62]. It can be clearly seen in Figure 14 that the high-frequency tail of the phase angle–frequency curve of X70 steel moves to a higher phase angle with the increase of NaCl content in soil corrosion environment. This indicates that more corrosion products are produced on the surface of X70 steel as the sodium chloride content increases at 90 days and that the large amount of corrosion products combine with the soil slime particles to form a more compact layer of corrosion products, which hinders ion transfer during the reaction. However, the corrosion of metals in soil is a very complex process. As the corrosion reaction continues, due to the high physical adsorption of chloride ions, it will promote corrosion through an adsorption-dissolution process between the chloride ions and the metal atoms. This makes the corrosion reaction of X70 steel more intense and produces more corrosion products in corrosive environments with high sodium chloride content. The macroscopic corrosion morphology of the X70 steel after rust removal showed that the corrosion pits on the surface of the X70 steel were fewer in the specimens obtained from soil columns with almost no NaCl and soil columns with lower NaCl content. In contrast, the corrosion pits on the surface of the X70 steel specimens from soil columns with 1.0% NaCl content were larger and more numerous. To further illustrate the status of these corrosion pits and explore the severity of pitting corrosion on the X70 steel surface, three-dimensional morphology images of the corrosion pits at 90 days were obtained, as shown in Figure 15. In the figure are the lowest points of corrosion pits as the reference height 0 point to obtain the full picture of X70 steel surface corrosion pits, while the largest corrosion pit’s three-dimensional shape is enlarged and placed on the right side of the figure. The three-dimensional morphology of the X70 steel clearly demonstrated that, with increasing NaCl content in the corrosion system, the number and size of corrosion pits on the metal surface increased, which was consistent with the macroscopic image analysis.

The microscopic morphology of the X70 steel specimens shown in Figure 13 demonstrated that the X70 micro-samples from soil columns with three different NaCl concentrations had cracks or pore defects. This was conducive to accelerating the corrosion reaction [63]. In this corrosion system, erosive fluid entered these cracks and directly contacted the metal matrix contact. As the reaction proceeded, the concentration of dissolved oxygen within these cracks decreased, leading to oxygen-deficient areas within the cracks. The cracks outside the corrosion system, therefore, became relatively oxygen-rich areas. This oxygen concentration difference led to a macroscopic corrosion cell effect. The interior of this crack was the anode of the corrosion cell due to its lower concentration of oxygen and more negative potential, while the metal outside the crack was the cathode of the corrosion cell due to the relatively high concentration of oxygen and more positive potential. This macroscopic corrosion cell effect accelerated the corrosion reaction of the X70 steel inside the crack. This phenomenon was more significant for the soil columns with high Cl^−^ concentrations. This was because the higher Cl^−^ concentration in the pore solution of the soil particles in these columns caused the potential to become more negative, leading to a higher corrosion rate.

**Figure 16:**
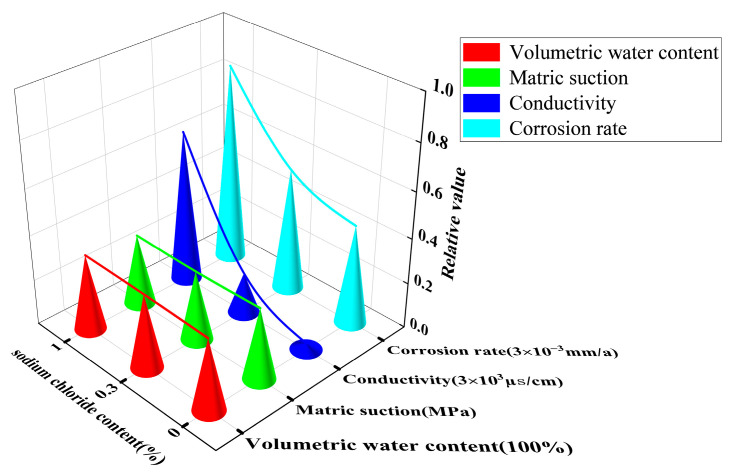
Variation trend of corrosion rate of X70 steel sheet samples with soil volumetric water content, electrical conductivity, and matrix suction at 90 days’ age at height position 12^#^ of soil column with different NaCl content.

As shown in Figure 16, at height position 12^#^ in the soil column, the change in volumetric water content and matrix suction between the soil columns with different NaCl content tended to be smooth. This mainly showed that the corrosion rate increased with increasing salt content (i.e., the corrosion rate increased with increasing soil conductivity). In summary, this analysis demonstrated that the NaCl content of the soil columns played a decisive role in determining the corrosion rates of the X70 steel specimens located at the same height positions.

**Figure 17:**
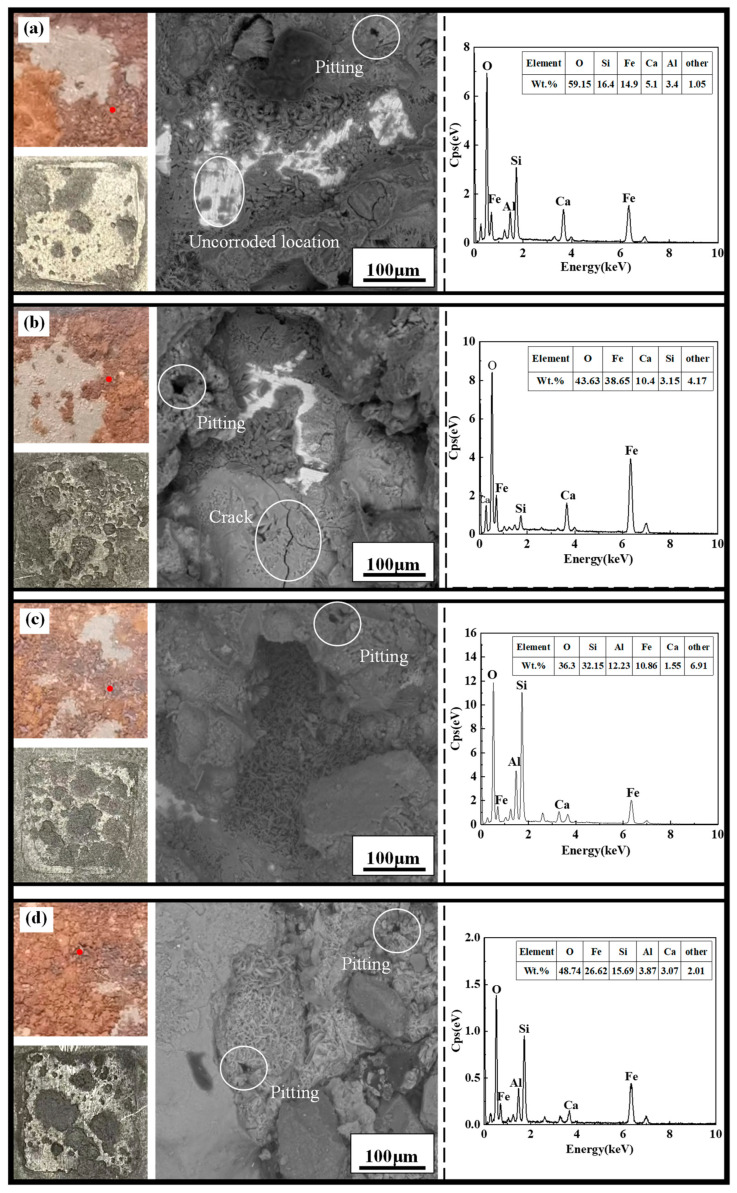
Macroscopic and microscopic corrosion morphology and elemental analysis results of X70 steel under capillary action in saline soils with a NaCl content of 0.3%: (**a**) 12^#^, (**b**) 27^#^, (**c**) 42^#^, and (**d**) 57^#^.

**Figure 18:**
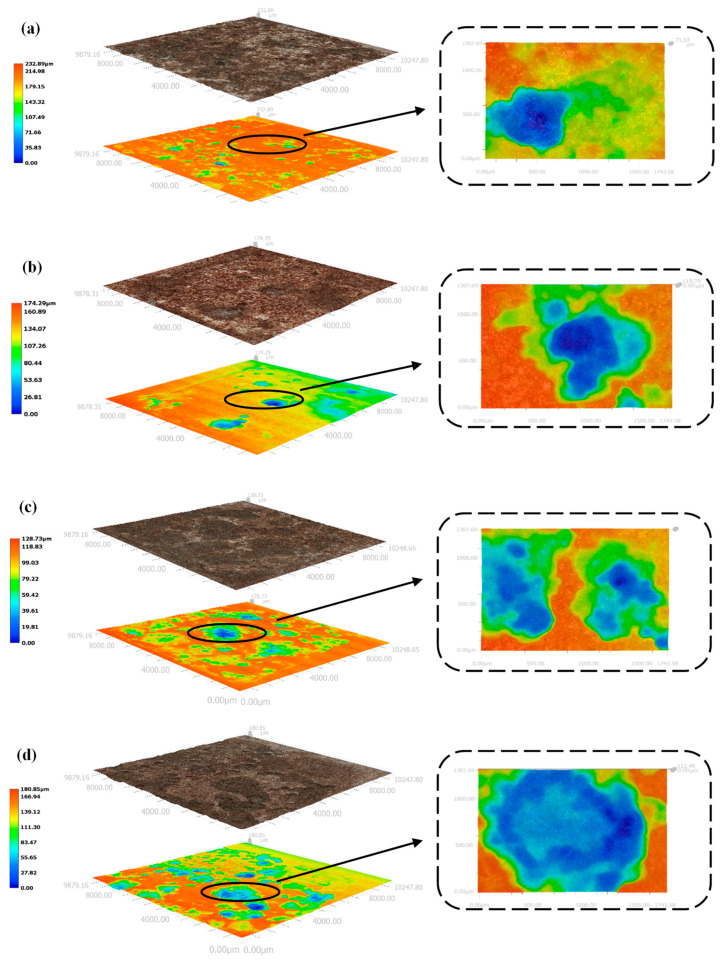
Three-dimensional morphological images of corrosion pits on the surface of X70 steel in soil column with 0.3% NaCl content: (**a**) 12^#^, (**b**) 27^#^, (**c**) 42^#^, and (**d**) 57^#^.

**Figure 19:**
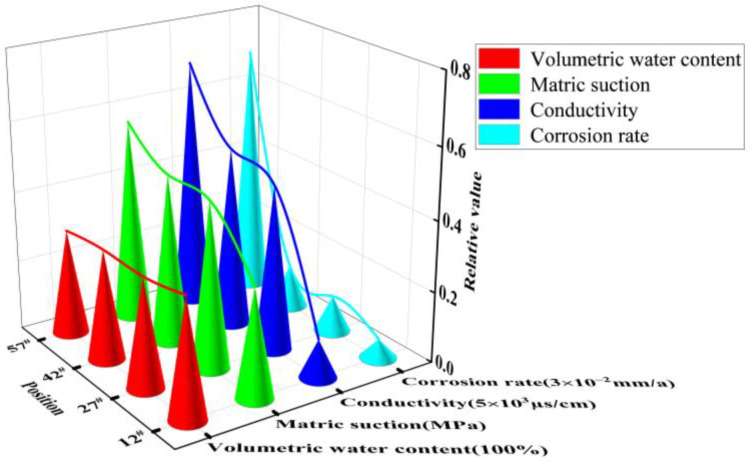
Variation trends of corrosion rate, soil volumetric water content, electrical conductivity, and matrix suction of X70 steel plate specimens aged 90 day at four height positions of 12^#^, 27^#^, 42^#^, and 57^#^ with a soil column with NaCl content of 0.3%.

The macroscopic image of the X70 steel corrosion products at 90 days shown in Figure 17 demonstrates that the X70 steel specimen buried at height position 57^#^ in the soil column exhibited a significantly larger corroded area compared with the specimens buried at positions 12^#^, 27^#^, and 42^#^. The corrosion pits on the surface of the X70 steel specimen buried at position 57^#^ were large and deep. In contrast, the X70 steel specimens buried at the other three positions exhibited less severe corrosion pits. As shown in Figure 18, the three-dimensional morphology of the X70 steel corrosion pits at 90 days also showed the same pattern. The reason for this trend is illustrated in Figure 19. On the one hand, after capillary water stabilization, the NaCl in the soil columns accumulated at the highest position (57^#^) with the migration of the capillary water. This led to a higher ion concentration and higher conductivity at position 57^#^. Thus, X70 steel corrosion at this location was accelerated, leading to a larger corrosion product area as well as large and deep corrosion pits. On the other hand, the final stabilized volume water content at different heights in the soil column after capillary stabilization tended to decrease with increasing distance from the bottom of the column. This led to increasing matrix suction with increasing distance from the bottom of the column. Higher matrix suction near the X70 steel sheet specimens led to an increased difference between the water pressure below the curved liquid level at the gas–liquid interface at the X70 steel interface and the atmospheric pressure. Thus, the moisture and ions in the soil were more easily adsorbed by the droplets on the surface of the X70 steel specimens, which facilitated the corrosion reaction. The deposition of corrosion products formed at different times during the corrosion process resulted in the significant delamination of some corrosion product aggregates [64]. As shown by the macroscopic image in Figure 17, the loose corrosion products on the surface of the X70 steel specimen combined with the fine sticky particles in the soil. The layering of corrosion products was more visible in the corresponding microscopic images, which showed a thin, dense inner layer of corrosion products and a thick, porous outer layer with a large number of adsorbed clay particles. The presence of this corrosion product layer inhibited uniform corrosion. Although some oxides in the corrosion products formed a protective corrosion product layer on the surface of the X70 steel sample, the uneven and non-compact corrosion product layer exhibited a number of cracks. These cracks led to the easy diffusion of the corrosive medium to the bottom of the corrosion product layer. Thus, a blocking corrosion battery effect was generated, which promoted pitting. In addition, the NaCl in the soil column accumulated at height position 57^#^ with the rising capillary water. This intensified the defects in the corrosion product layer of the X70 steel specimen at position 57^#^. At the same time, these significant corrosion product layer defects meant that the corrosive medium was more easily transmitted and diffused through the layer, which aggravated the reaction.

Element analysis of the micro-corrosion morphology area shown in Figure 13 and Figure 17 demonstrated that the main elements of the corrosion products in the corrosion system were O and Fe. Thus, iron oxide was the main corrosion product in this corrosion system. Element analysis of some positions in Figure 13 and Figure 17 showed that the Ca and Si content of these positions was also high. This was because the soil used in this experiment was silty clay. Some of the fine clay particles in this soil was adsorbed on the surface of the corrosion products. The low Al content observed in the elemental analysis results potentially came from the sample test.

#### 3.4.2. Corrosion Mechanism of X70 Steel under Capillary Action

**Figure 20:**
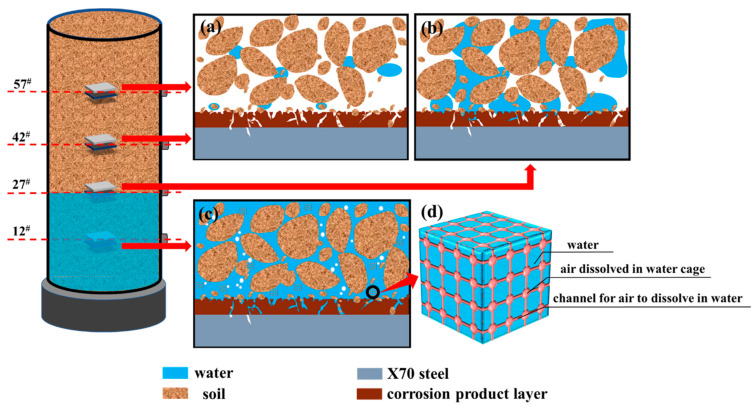
Morphological changes of solid-liquid-gas three-phase due to rising capillary water in Geo-experts one-dimensional soil column instrument X70 steel corrosion system:(**a**) locations above the capillary water wetting front, (**b**) location of the capillary water wetting front, (**c**) locations below the capillary water wetting front, (**d**) the air dissolved in the water is stored in the grid structure with many eyelets.

The changes in the solid-liquid-gas three-phase morphology of the X70 steel corrosion system due to the rise of the capillary water are shown in Figure 20. The unsaturated soil was a multi-phase mixture with three phases: a solid skeleton, the pore water, and gas. This did not take the shrinkage film into account. The pores between the soil particles were interconnected, forming a soil pore system. This pore system provided the channels through which capillary water rose in the unsaturated soils. The rise of the capillary water in the soil column was a process in which the liquid water changed on the metal surface.

As the capillary water rose, three different solid-liquid-gas three-phase morphology states were observed in the corrosive environments of the X70 steel specimens at four different height positions in the soil column. The solid-liquid-gas three-phase morphology in the corrosive environment of X70 steel specimens at locations above the capillary water wetting front is shown in Figure 20a. In this state, the water in the pores solely relied on the surface tension of the curved liquid surface of the gas–water interface. Thus, this water was held in small voids in some narrow pore channels or in the solid particles located around the formed isolated water rings or corner points. This made the ion exchange and charge migration in the corrosion system more difficult, which was not conducive to the corrosion process. The three-phase solid-liquid-gas morphology in the corrosive environment of the X70 steel specimen at the location of the capillary water wetting front is shown in Figure 20b. This corrosive environment exhibited a higher corrosion rate, because the pore water and gas were connected in this corrosion system. The connected pore water provided good conditions for ion exchange and charge migration in the corrosion system, while the connection of the gas in the soil pores provided a large amount of oxygen for the corrosion reaction to take place. Thus, the corrosion of the X70 steel was promoted. The three-phase solid-liquid-gas morphology in the corrosive environment of the X70 steel specimens at locations below the capillary water wetting front is shown in Figure 20c. As the capillary water rose, the water content of the contaminated soil corrosion system below the capillary water wetting front continued to increase. The water eventually occupied almost all the pores. At this time, oxygen from the air dissolved in the water was required for the X70 steel corrosion reaction. These water molecules were arranged in a grid structure with many eyelets, forming ‘cages’ to hold the air, as shown in Figure 20d.

**Figure 21:**
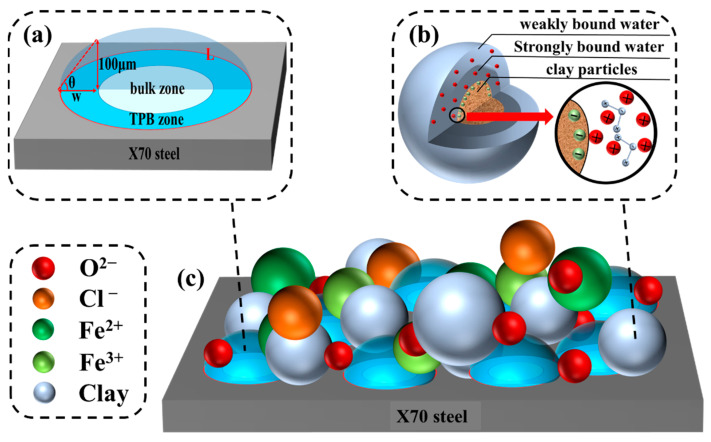
Schematic illustration of interfacial corrosion between X70 steel and chlorine-containing pore solution: (**a**) geometry of the TPB and volumetric region of electrolyte droplets on a flat metal surface, (**b**) cohesive soil particle structure and its ion exchange mode on the metal surface, (**c**) schematic diagram of each ion participating in the reaction on the metal surface.

Dissolution of the metal occurred in the anodic region (Equation (8)) and the depolarization of oxygen occurred in the cathodic region (Equation (9)). Droplets in the corrosion system were adsorbed on the metal surface. The electrochemical reaction zone under the liquid layer was divided into a bulk zone and a three-phase boundary zone, as shown in Figure 21a. When the electrolyte thickness was 0–100 μm (TPB zone), the average rate of oxygen diffusion was higher than that of the bulk solution. Therefore, the TPB zone played a more important role than the bulk zone in the cathodic oxygen reduction process [65]. At the same time, the presence of more fine sticky particles in this test corrosion system meant that the liquid on the metal surface was highly dispersed, which led to the existence of a large number of dispersed TPB areas, as shown in Figure 21c. This phenomenon significantly accelerated the cathodic oxygen reduction process and also led to higher X70 steel corrosion at the capillary water wetted front (i.e., the solid/liquid/gas three-phase interface). The intensification of the cathodic reaction led to the generation of many hydroxide ions, with ferrous and hydroxide ions further producing green ferrous hydroxide (Equation (10)). This ferrous hydroxide continued to react in the presence of oxygen and water to form insoluble ferric hydroxide (Equation (11)). Iron hydroxide is not very stable. Thus, the iron hydroxide continued to react in the corrosion system to produce more stable corrosion products: hydroxy iron oxide (Equation (12)), ferric oxide (Equation (13)), and ferric tetroxide (Equation (14)). These corrosion products combined with the fine sticky particles, which adhered to the surface of the X70 steel substrate to form a protective corrosion product layer on the metal substrate. As the corrosion reaction proceeded, a dense corrosion product passivation film was formed on the metal substrate surface. This film hindered the continuation of the corrosion. However, Cl^−^ has a small ionic radius. Thus, Cl^−^ could more easily penetrate the passivation film compared with other ions under the action of diffusion and electric field through the defects in the film as well as the metal reaction (Equations (15) and (16)). This ensured the continuation of the corrosion reaction. In addition, moisture in the soil penetrated the soil–steel contact interface when the X70 steel surface was in contact with wet clay. The X70 steel and this moisture generated electric double-layer capacitance at the solid–liquid two-phase interface. This led to an electrical potential difference on the surface of the X70 steel, which formed a primary cell that caused localized corrosion. Thus, the corrosion rate of the X70 steel was enhanced. The structure of the clay particles and their charge transfer patterns are shown in Figure 21b.

(8) $$Fe-2e^{-}\to Fe^{2+}$$

(9) $$\frac{1}{2}O_{2}+H_{2}O+2e^{-}\to 2OH^{-}$$

(10) $$Fe^{2+}+2OH^{-}\to Fe{\left(OH\right)}_{2}$$

(11) $$2Fe{\left(OH\right)}_{2}+\frac{1}{2}O_{2}+H_{2}O\to 2Fe{\left(OH\right)}_{3}$$

(12) $$Fe{\left(OH\right)}_{3}\to FeOOH+H_{2}O$$

(13) $$2Fe{\left(OH\right)}_{3}\to Fe_{2}O_{3}\cdot 3H_{2}O\to Fe_{2}O_{3}+3H_{2}O$$

(14) $$2FeOOH+Fe^{2+}\to Fe_{3}O_{4}+2H^{+}$$

(15) $$Fe^{3+}\left(in\;passivation\;film\right)+3Cl^{-}\to FeCl_{3}$$

(16) $$FeCl_{3}\to Fe^{3+}\left(in\;electrolyte\right)+3Cl^{-}$$

## 4. Conclusions

In this work, the electrochemical corrosion behavior of X70 steel in saline soils under capillary action was simulated by a Geo-experts one-dimensional soil column meter, and the following conclusions were obtained:

(1) The final stable water content of the soil column decreased with increasing height, and the rate of the capillary water rise was accelerated by the NaCl content of the soil, which promoted capillary action. The transport of salts in the soil lagged relative to the transport of water. Height position 12^#^ in the soil column was used as a dividing interface. The soil conductivity above this position showed an increasing trend with rising capillary water.
(2) The corrosion current density and corrosion rate of the X70 steel specimens at the same height positions in the soil column increased with increasing NaCl content. The corrosion behavior of X70 steel at different heights in the same soil column was significantly influenced by the transport of water and salt caused by the rise of the capillary water. The rise of the capillary wetting front position led to a solid/liquid/gas three-phase interface with the X70 steel specimens that enhanced the corrosion behavior. In addition, the accumulation of salts at a specific location also enhanced the X70 steel corrosion rate at that location.
(3) The electrochemical impedance spectra of X70 steel in a capillary water environment exhibited a superposition of a bicircular arc with two time constants and a straight line representing mass transfer diffusion in the low-frequency region. The radius of the high-frequency capacitive arc resistance in the Nyquist plots decreased with increasing NaCl content in the soil column. Capillary pore channels below the capillary water wetting front were easily occupied by the solution. This led to an anoxic corrosion environment that reduced the X70 steel corrosion kinetics.
(4) Higher NaCl content in the soil column led to a larger area covered by corrosion products, a greater number of corrosion pits, and deeper pits on the X70 steel specimen surfaces. After the capillary water was stabilized for a period of time, the area of these pits on the surface of the X70 steel specimen at height position 57^#^ was significantly larger than that at other height positions. The pits at position 57^#^ were also deeper. This was due to the combined effect of the changes to a number of factors caused by the rise of capillary water.

## Figures and Tables

**Table 1:** The main chemical composition of X70 pipeline steel (wt.%).

Chemical Composition	C	Mn	Si	P	S	Nb	Cu	Cr	Ni	Mo	Ti
Values/(%)	0.050	1.540	0.190	0.009	0.001	0.070	0.230	0.200	0.200	0.190	0.02

**Table 2:** Basic physical parameters of the soil.

Parameters	Values
natural water content/(%)	12.24
original dry density/(g·cm^−3^)	1.490
maximum dry density/(g·cm^−3^)	1.810
optimum moisture content(%)	20.80
liquid limit/(%)	30.30
plastic limit/(%)	18.80
plastic index	11.50

**Table 3:** Proportion of soil samples with different NaCl content.

Water Content/(%)	NaCl Content/(%)	Dry Soil Quality/(g)	NaCl Quality/(g)	Water Quality/(g)
12.24	0	18,000	0	1689.3
	0.3	18,000	54.0	1689.3
	1.0	18,000	180.0	1689.3

**Table 4:** Electrochemical corrosion parameters of X70 steel at 12^#^ height.

Time/(Day)	NaCl Content/(%)	Ba/(mV/dec)	Bc/(mV/dec)	R_P_/(Ω/cm^2^)	Icorr/(A/cm^2^)	Ecorr/V	Corrosion Rate/(mm/a)
14	0.0%	295.61	168.37	9.64 × 10^5^	3.3826 × 10^−8^	−0.4848	0.3979 × 10^−3^
	0.3%	374.03	168.30	8.19 × 10^5^	6.0887 × 10^−8^	−0.5913	0.7162 × 10^−3^
	1.0%	730.79	195.88	6.85 × 10^5^	7.8136 × 10^−8^	−0.6645	0.9191 × 10^−3^
28	0.0%	166.12	120.50	8.90 × 10^5^	4.0271 × 10^−8^	−0.6224	0.4737 × 10^−3^
	0.3%	329.91	330.07	8.36 × 10^5^	6.0692 × 10^−8^	−0.6614	0.7139 × 10^−3^
	1.0%	238.77	180.14	6.11 × 10^5^	1.1094 × 10^−7^	−0.4396	1.3049 × 10^−3^
45	0.0%	158.14	114.31	9.92 × 10^5^	2.8260 × 10^−8^	−0.5743	0.3324 × 10^−3^
	0.3%	1731.4	263.48	5.26 × 10^5^	1.7347 × 10^−7^	−0.6943	2.0404 × 10^−3^
	1.0%	742.85	217.10	4.80 × 10^5^	1.8616 × 10^−7^	−0.6034	2.1896 × 10^−3^

**Table 5:** Electrochemical corrosion parameters of X70 steel in soil with 0.3% sodium chloride content.

High Position	Time/ (Day)	Ba/(mV/dec)	Bc/(mV/dec)	R_P_/(Ω/cm^2^)	Icorr/(A/cm^2^)	Ecorr/V	Corrosion Rate/(mm/a)
12^#^	14	374.03	168.30	8.19 × 10^5^	6.0887 × 10^−8^	−0.5913	0.7162 × 10^−3^
	28	329.91	330.07	8.36 × 10^5^	6.0692 × 10^−8^	−0.6614	0.7139 × 10^−3^
	45	1731.4	263.48	5.26 × 10^5^	1.7347 × 10^−7^	−0.6942	2.0404 × 10^−3^
27^#^	14	184.58	139.67	1.33 × 10^6^	1.9443 × 10^−8^	−0.5745	0.2287 × 10^−3^
	28	313.66	256.77	6.52 × 10^5^	1.0495 × 10^−7^	−0.8163	1.2344 × 10^−3^
	45	441.71	184.82	8.06 × 10^5^	4.5026 × 10^−8^	−0.7477	0.5296 × 10^−3^
42^#^	14	454.41	327.71	8.27 × 10^6^	6.5365 × 10^−9^	−0.5720	0.0769 × 10^−3^
	28	446.85	247.65	2.72 × 10^6^	9.2309 × 10^−9^	−0.6677	0.1086 × 10^−3^
	45	403.99	222.72	6.32 × 10^6^	8.1436 × 10^−9^	−0.6921	0.0959 × 10^−3^
57^#^	14	296.26	232.05	7.81 × 10^5^	4.5504 × 10^−8^	−0.6889	0.5352 × 10^−3^
	28	305.78	169.84	7.56 × 10^5^	4.2272 × 10^−8^	−0.8030	0.4972 × 10^−3^
	45	202.00	186.62	6.02 × 10^5^	1.2525 × 10^−7^	−0.4773	1.4732 × 10^−3^

**Table 6:** Equivalent circuit parameters of X70 steel in soil.

Time/ (Day)	NaCl Content/ (%)	R_s_/ (Ω·cm^2^)	C_cp_/ (F·cm^−2^)	R_cp_/ (Ω·cm^2^)	C_s_/ (F·cm^−2^)	R_ct_/ (Ω·cm^2^)	W1-R	W1-T	W1-P
14 d	0.0%	203.8	8.45 × 10^−10^	2733	9.23 × 10^−8^	1225	1.66 × 10^6^	191.9	0.6185
	0.3%	249.1	1.10 × 10^−9^	1118	2.57 × 10^−7^	586.6	3.68 × 10^5^	15.84	0.6923
	1.0%	148.6	3.59 × 10^−6^	54.56	5.58 × 10^−6^	478.5	3.38 × 10^5^	16.54	0.7302
28 d	0.0%	395.6	1.16 × 10^−9^	2035	1.28 × 10^−7^	1558	1.69 × 10^6^	145.0	0.6878
	0.3%	291.2	1.31 × 10^−9^	992.0	2.44 × 10^−7^	568.4	1.96 × 10^5^	6.941	0.7036
	1.0%	271.2	2.86 × 10^−9^	615.4	4.61 × 10^−6^	386.8	2.09 × 10^4^	1.303	0.4609
45 d	0.0%	314.2	1.06 × 10^−9^	2209	1.26 × 10^−7^	1187	5.48 × 10^5^	33.74	0.6918
	0.3%	252.5	1.20 × 10^−9^	1021	2.60 × 10^−7^	163.0	2.60 × 10^5^	11.42	0.7139
	1.0%	224.2	1.08 × 10^−9^	990.3	1.91 × 10^−6^	114.0	1.35 × 10^5^	8.428	0.7065

**Table 7:** Equivalent circuit parameters of X70 steel in soil with 0.3% sodium chloride content.

High Position	Time/ (Day)	R_s_/ (Ω·cm^2^)	C_cp_/ (F·cm^−2^)	R_cp_/ (Ω·cm^2^)	C_s_/ (F·cm^−2^)	R_ct_/ (Ω·cm^2^)	W1-R	W1-T	W1-P
12^#^	14 d	249.1	1.10 × 10^−9^	1118	2.57 × 10^−7^	586.6	3.68 × 10^6^	15.84	0.6923
	28 d	291.2	1.30 × 10^−9^	992.0	2.44 × 10^−7^	568.4	1.96 × 10^6^	6.941	0.7036
	45 d	252.5	1.20 × 10^−9^	1021	2.60 × 10^−7^	163.0	2.60 × 10^6^	11.42	0.7139
27^#^	14 d	175.6	1.19 × 10^−6^	29.77	1.81 × 10^−7^	493.9	2.95 × 10^6^	19.39	0.6933
	28 d	169.2	4.30 × 10^−6^	30.14	2.58 × 10^−6^	473.8	1.99 × 10^6^	4.939	0.7162
	45 d	161.7	3.52 × 10^−6^	71.96	4.07 × 10^−6^	661.1	4.27 × 10^6^	10.58	0.6986
42^#^	14 d	414.5	4.22 × 10^−9^	2804	7.45 × 10^−10^	846.6	2.81 × 10^7^	0.303	0.2811
	28 d	618.6	1.21 × 10^−9^	6170	2.85 × 10^−9^	598.3	3.79 × 10^7^	98.42	0.3096
	45 d	388.0	8.92 × 10^−9^	4455	1.27 × 10^−8^	631.2	1.32 × 10^7^	7.561	0.5210
57^#^	14 d	232.2	3.39 × 10^−6^	106.8	1.77 × 10^−7^	514.9	1.47 × 10^6^	4.994	0.7032
	28 d	214.0	4.07 × 10^−6^	96.72	2.71 × 10^−7^	695.1	3.90 × 10^6^	14.47	0.7535
	45 d	224.4	4.12 × 10^−6^	64.16	6.03 × 10^−6^	1070	8.84 × 10^5^	8.541	0.6606

## Data Availability

Data presented in this study are available on request from the corresponding authors.
